# Supplementary material for: Evanescent scattering imaging of single protein binding kinetics and DNA conformation changes
Source: Nat Commun. 2022 Apr 28;13:2298. doi: 10.1038/s41467-022-30046-8 (PMC9051210; doi:10.1038/s41467-022-30046-8)
Supplement: Supplementary file 1 — Supplementary information [file 41467_2022_30046_MOESM1_ESM.pdf]

Supplementary Information

Evanescent Scattering Imaging of Single Protein Binding Kinetics and DNA Conformation Changes

Pengfei Zhang[1,§], Lei Zhou[2,§], Rui Wang[1], Xinyu Zhou[1,3], Jiapei Jiang[1,3], Zijian Wan[1,4], Shaopeng Wang[1,3*]

1. Biodesign Center for Bioelectronics and Biosensors, Arizona State University, Tempe, AZ, USA

2. Center for Biological Physics, School of Molecular Sciences, Department of Physics, Arizona State University, Tempe, AZ, USA

3. School of Biological and Health Systems Engineering, Arizona State University, Tempe, AZ, USA

4. School of Electrical, Energy and Computer Engineering, Arizona State University, Tempe, AZ, USA

§ These authors contributed equally.

* Corresponding author contact email: Shaopeng.Wang@asu.edu

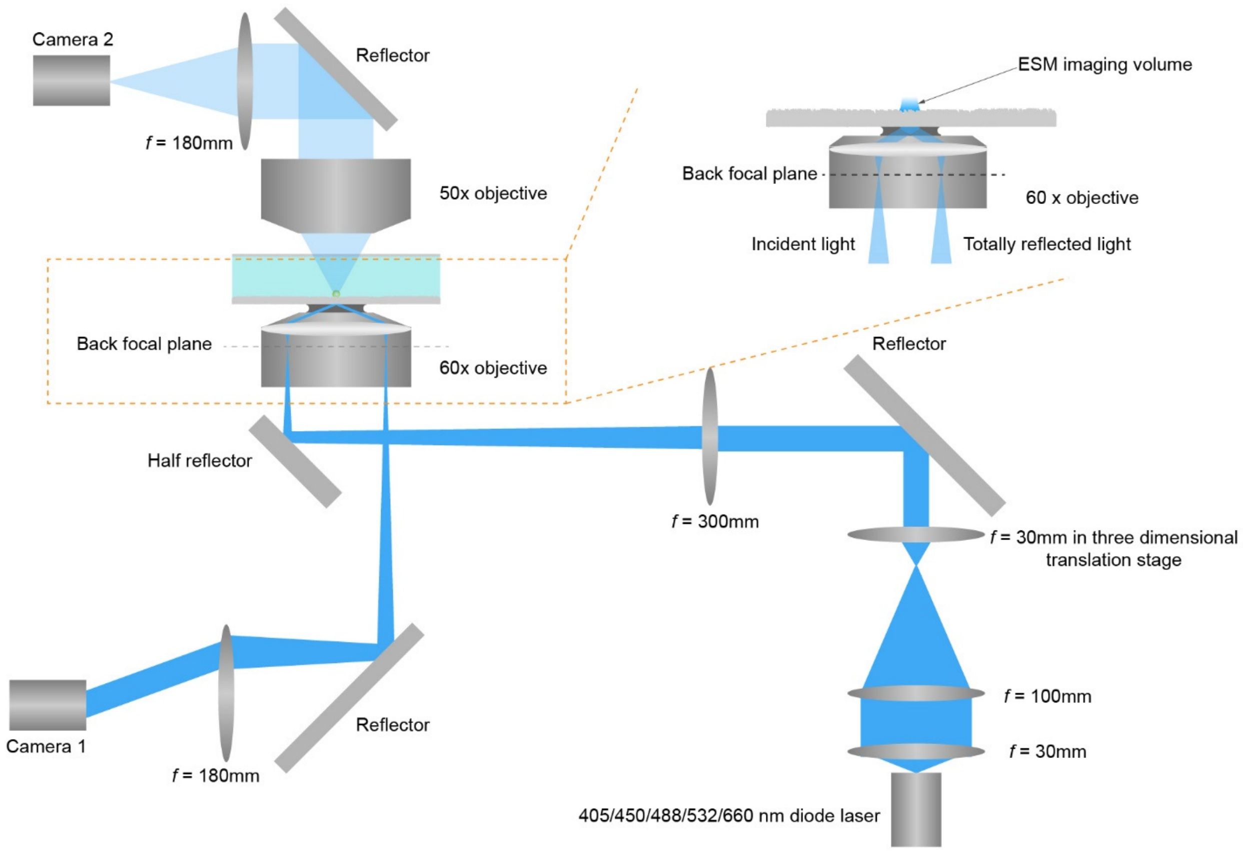

Supplementary Figure 1

Schematic diagram of the optical setup for evanescent scattering microscopy (ESM). Light from the diode laser is conditioned and focused onto the back focal plane of a 60x objective (NA=1.49). Then the collimated laser beam was directed onto a cover glass mounted on the objective via refractive index match oil. Light reflected from the gold-coated glass slide is detected by camera 1 (MQ013MG-ON, XIMEA), which is equipped with an optical attenuator (ND30A, Thorlabs, Newton, NJ) to avoid overexposure. The reflection light is used to find the 60x objective focus. The incident light angle is adjusted to 65° using a three-dimensional translation stage (XR25P-K2, Thorlabs) to achieve total internal reflection (TIR). The incident light intensity is 60 kW cm^{-2} or less. Light scattered from the glass surface is collected by a 50x objective (NA=0.42) and detected by camera 2 (MQ003MG-CM, XIMEA) placed on top of the samples. A thin cover glass constructed flow cell constraining ~50 µm channel height was employed for sample delivery (Nat Methods 17, 1010–1017 (2020)). An 80-mW laser diode (PL450B, Thorlabs, Newton, NJ, US) is used as the light source to provide the incident light with central wavelength at 450 nm for single protein imaging. Coherent OBIS FP 405 LX, OBIS FP 488LS, OBIS FP 532LS, and OBIS FP 660 LX lasers were used as the light source to provide the incident light with central wavelength at 405, 488, 532, and 660 nm to explore the effect of incident wavelength on image intensity.

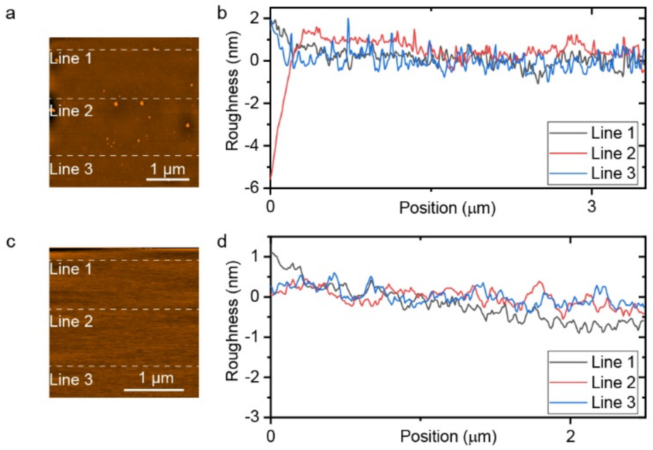

Supplementary Figure 2

Surface roughness of cover glass.

a, Atomic Force Microscopy image of an uncleaned cover glass. **b**, Profiles of marked lines in (a). **c**, Atomic Force Microscopy image of a cover glass after ultrasonic cleaning with 1:1 ethanol and acetone mixture. **d**, Profiles of marked lines in (c).

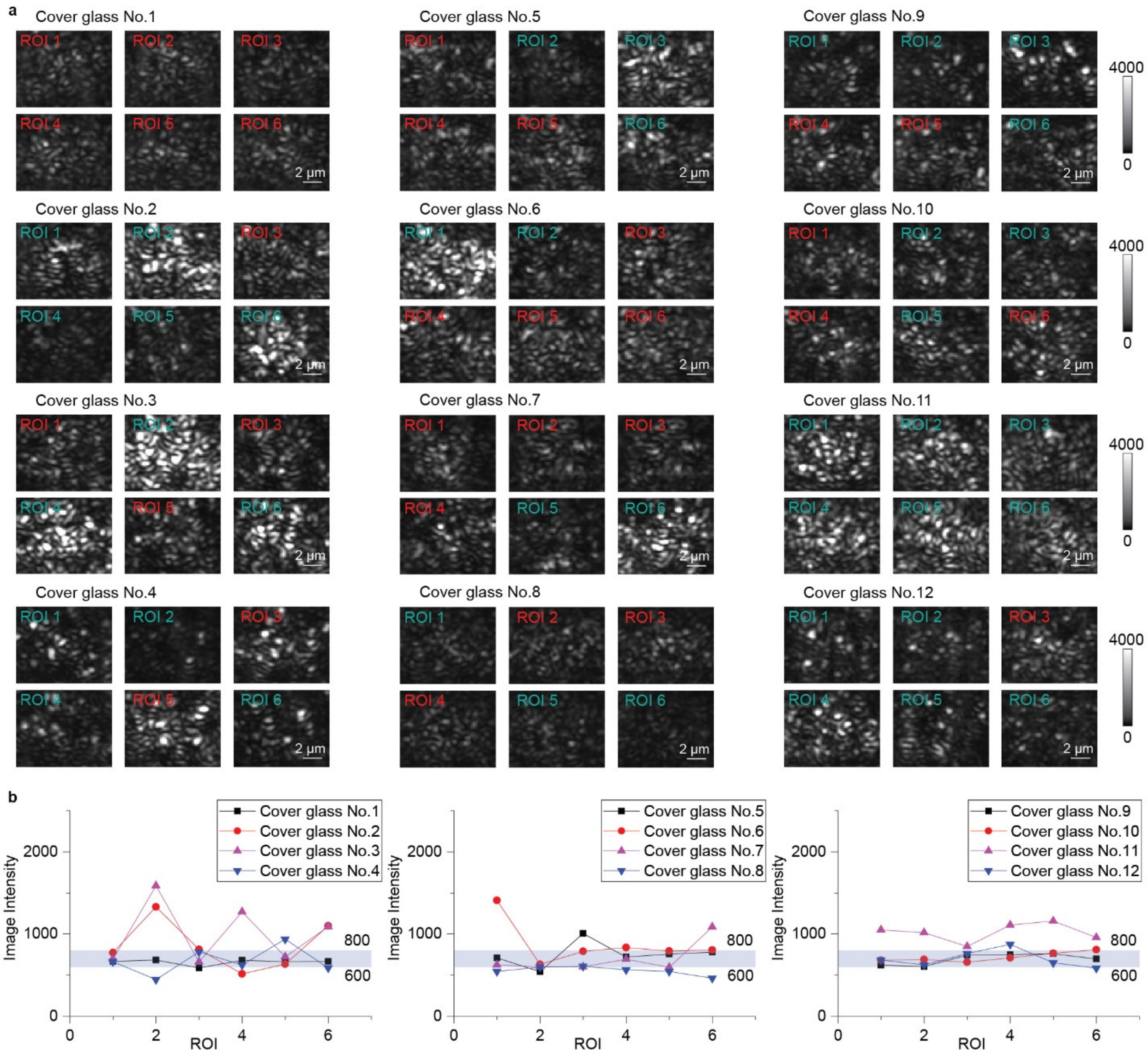

Supplementary Figure 3

ESM images of surface roughness of cover glass.

a, ESM images of surface roughness on different regions of interest (ROI) on 12 pieces of cover glasses. The cover glasses were randomly picked from a commercial batch. The images show that some cover glasses have uniform surface roughness profiles, while others have uneven roughness profiles. The experiments were repeated three times with similar results. **b**, Mean images intensities in different ROIs. The results show that at least one region in most cover glasses has roughness profiles with mean ESM image intensity between 600 and 800 grayscales. These regions (marked with a red frame in a) were selected to ensure measurement reproducibility. Incident wavelength: 450 nm. Incident intensity: 60 kW cm^{-2}. Camera exposure time: 5 ms.

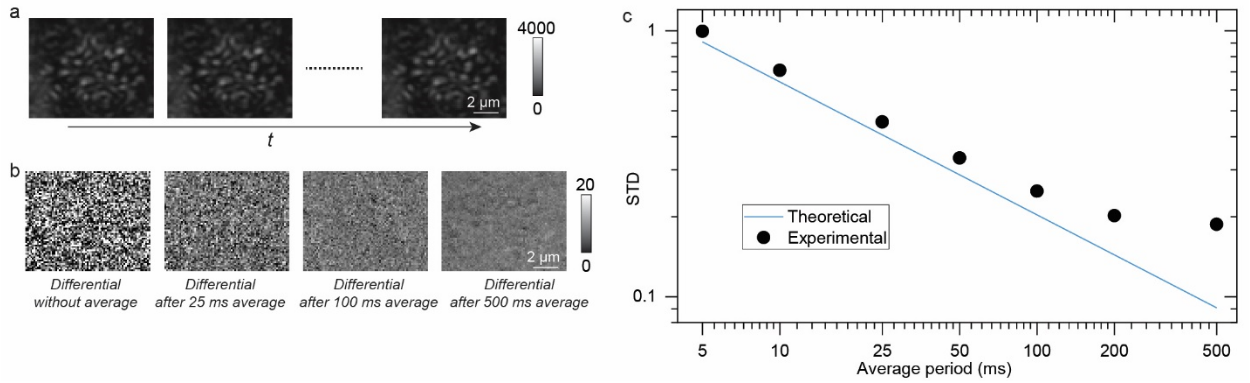

Supplementary Figure 4

Suppressing the shot noise with image average.

a, The raw image sequence of a bare cover glass was recorded at 200 frames/s for 30 s. **b,** Differential images were achieved by subtracting a previous frame from each frame after averaging the images with different periods. Due to the shot noise, which is intrinsic and scales with the square root of the photon number (https://en.wikipedia.org/wiki/Shot_noise), the background is not zero but decreases against the average period within 100 ms. The background for the differential image after the 500 ms average becomes large again because of the system mechanical drift. **c,** Comparison of theoretical shot noise and experimental noise with different average periods, confirming that the shot noise is dominant for the average period below 100 ms. Incident light intensity, 60 kW cm^{-2}. Exposure time, 5 ms.

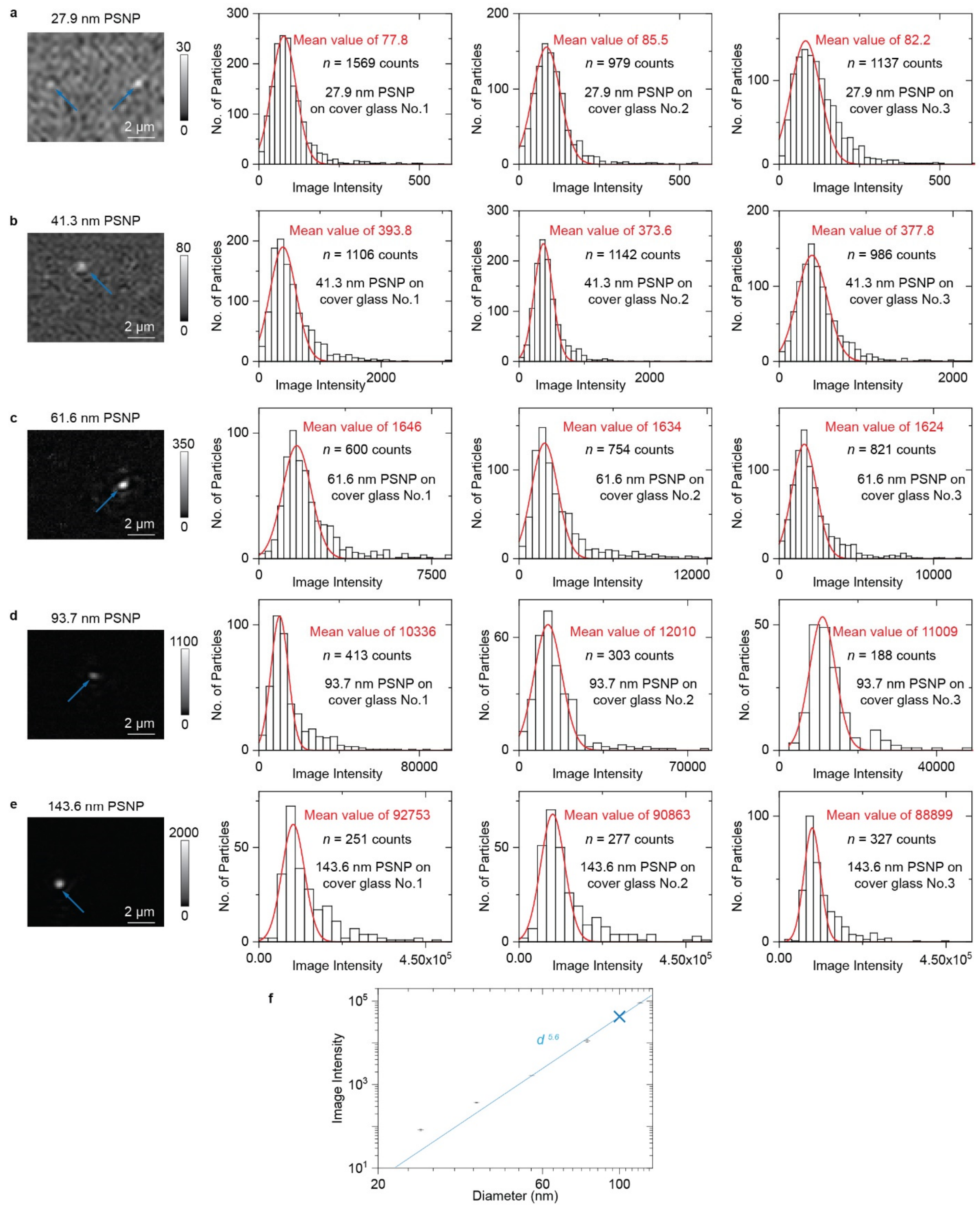

Supplementary Figure 5

a-e, ESM images and image intensity histograms of 27.9 nm (a), 41.3 nm (b), 61.6 nm (c), 93.7nm (d), and 143.6 nm (e) polystyrene nanoparticles (PSNP), where the solid red curves are Gaussian fittings. Each measurement was repeated on three different cover glasses. Incident intensity: 2 kW cm^{-2}. Camera exposure

time: 5 ms for 27.9 nm, 2 ms for 41.3 nm and 61.6 nm, 1 ms for 93.7 nm, 0.2 ms for 143.6 nm. The image intensity was normalized with an incident intensity of 60 kW cm^{-2} and a camera exposure time of 5 ms. The arrows indicate the position where the nanoparticle binds. The histogram width mainly results from the nanoparticle heterogeneity. The dynamic light scattering results show that the 27.9 nm, 41.3 nm, 61.6 nm, 93.7nm, and 143.6 nm PSNP have the diameter distribution width of 5.4 nm, 8.1 nm, 12.8 nm, 15.4 nm, and 15.6 nm, respectively. The experiments were repeated three times with similar results. **f,** ESM image intensity versus particle diameter. Horizontal lines represented the mean values, and the error bars were small, indicating good ESM measurement reproducibility. The effective particle diameter as discussed in Supplementary Note 1 was employed for the fitting. The cross sign indicates the image intensity of the PSNP with an effective diameter of 100 nm, which is used for studying the relationship between image intensity and incident wavelength shown in Figure 1g. The sample sizes are presented in a-e, and the counts come from independent nanoparticles. The centers and error bars represent the mean values and standard deviations achieved from the image intensities determined by three independent measurements.

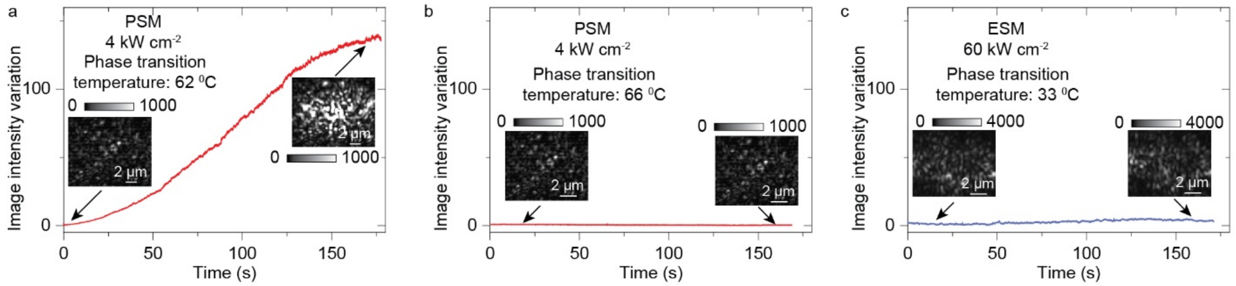

Supplementary Figure 6

Compare the light induced heating effect between PSM and ESM by monitoring the temperature-responsive phase transition of polymer.

Some polymer solutions will produce large vesicles when the solution temperature is higher than the phase transition temperature (J. Phys. Chem. B 2007, 111, 6, 1262–1270). The 0.236 g/L polyethylene oxide (PEO) dissolved in 300 mM NaH_2PO_4 and 240 mM NaH_2PO_4 aqueous solutions have the phase transition temperature at 62 °C and 66 °C, respectively. Phase transition was observed when flowing PEO in 300 mM NaH_2PO_4 solution onto the gold film surface of PSM (Supplementary Figure 6a), and not observed when flowing PEO in 240 mM NaH_2PO_4 solution (Supplementary Figure 6b). This indicates the PSM sensor surface temperature is between 62°C and 66 °C under the incident intensity of 4 kW cm^{-2}. This is close to the upper temperature limit for protein detection (Journal of Pharmaceutical and Biomedical Analysis 2020, 189, 113399), so it is hard to increase the incident intensity further. In contrast, phase transition was not observed on the glass surface of ESM under the incident intensity of 60 kW cm^{-2} when flowing 1 g/L cellulose in 300mM Na_2HPO_4 aqueous solution with a phase transition temperature of 33 °C. This indicates a much smaller heating effect on the glass surface than the gold surface, thus allowing the ESM to employ the incident intensity of 60 kW/cm^2, which is ~20 times higher incident intensity than reported value for PSM (Nat Methods 17, 1010–1017 (2020)). Camera exposure time is 0.1 ms for PSM, and 5 ms for ESM. A detailed study of the heating effect will be published in an upcoming article.

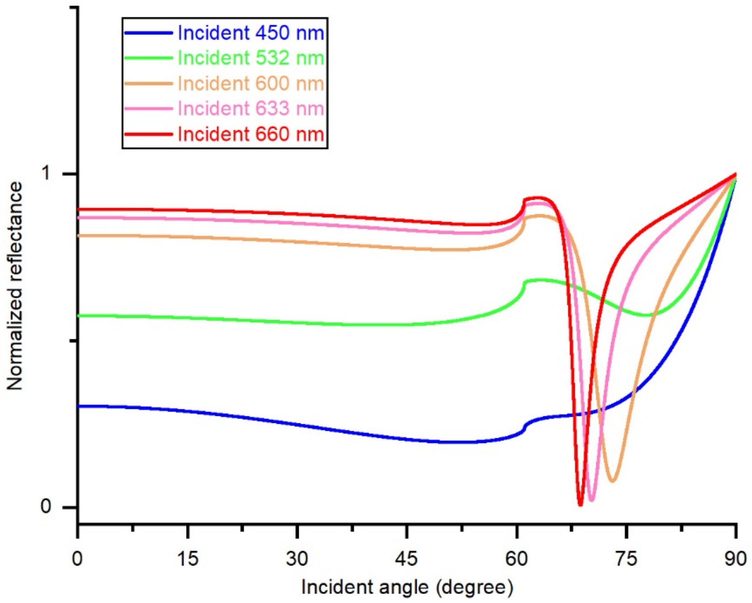

Supplementary Figure 7

Simulated surface plasmon resonance (SPR) curve for the gold-water interface under different incident wavelengths.

Winspall data analysis software (http://res-tec.de/downloads.html) is used to simulate SPR curves under different wavelengths. The gold refractive index is 1.52 + 1.96i, 0.48 + 2.36i, 0.24 + 3.23i, 0.19+ 3.59i, and 0.17+3.87i under incident wavelengths of 450 nm, 532 nm, 600 nm, 633 nm, and 660 nm, respectively. BK7 cover glass has a refractive index of ~1.52 under visible light (refractiveindex.info). Gold film thickness is set at 50 nm. The simulation shows that the SPR cannot be observed with blue and green incident light, and the SPR angle is ~75° under the incident wavelength of 600 nm. The commonly used immersion oil objective has a numerical aperture of 1.49, corresponding to the largest incident angle of ~78.6°. Thus, the SPR condition is hard to be achieved with an incident wavelength of 600 nm to construct PSM, and PSM usually requires the incident wavelength longer than 600 nm. In contrast, the BK7 cover glass and water have a stable refractive index over the entire visible light range, so ESM allows a wide incident wavelength range.

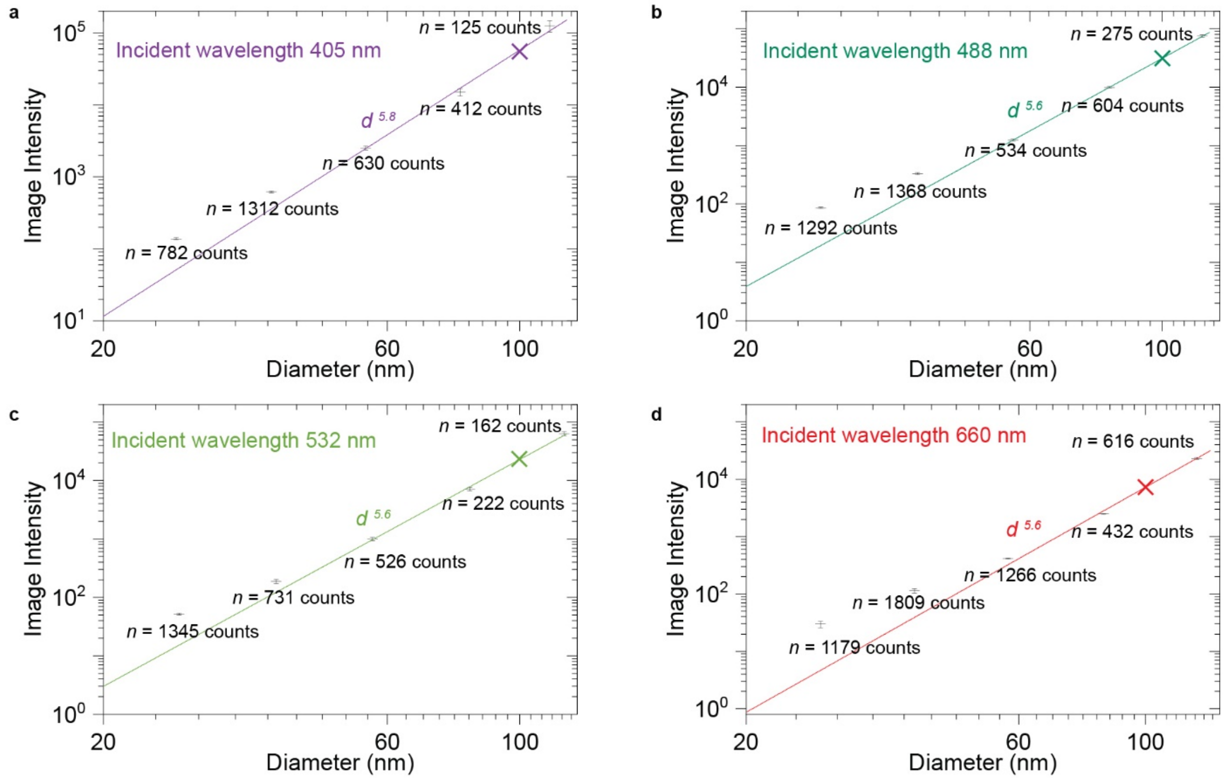

Supplementary Figure 8

ESM image intensity versus particle diameter under the incident wavelengths of 405 nm (a), 488 nm (b), 532 nm (c), and 660 nm (d). 27.9 nm, 41.3 nm, 61.6 nm, 93.7nm, and 143.6 nm polystyrene nanoparticles were measured on three different cover glasses. Horizontal lines represented the mean values, and the error bars were small, indicating good ESM measurement reproducibility. The effective particle diameter as discussed in Supplementary Note 1 was employed for the fitting. Incident intensity: 2 kW cm^{-2}. The image intensity was normalized with an incident intensity of 60 kW cm^{-2} and a camera exposure time of 5 ms. The cross sign indicates the image intensity of the PSNP with an effective diameter of 100 nm, which is used for studying the relationship between image intensity and incident wavelength shown in Figure 1g. The sample sizes are presented in the figures, and the counts come from independent nanoparticles. The centers and error bars represent the mean values and standard deviations achieved from the image intensities determined by three independent measurements.

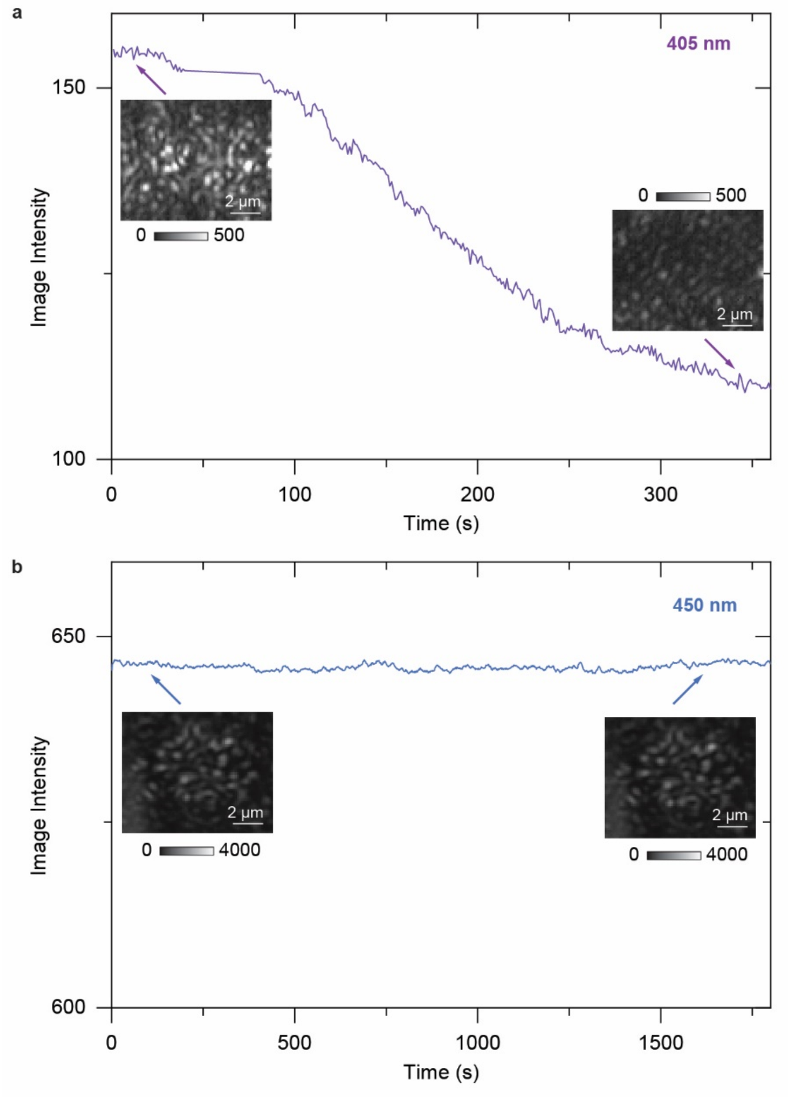

Supplementary Figure 9

Stability of the surface chemistry under the illumination of high intensity 405 nm and 450 nm light.

The cover glass was modified with NHS groups (Methods), and high concentration protein and 26 nm polystyrene nanoparticle solutions were flowed onto the sensor surface for binding. Then, the cover glass was illuminated by the 405 nm (a), and 450 nm (b) laser with an incident intensity of 60 kW cm^{-2}. For the incident wavelength of 405 nm, the analytes will leave the surface within about 5 minutes (300 s), whereas the surface chemical state is stable under the incident wavelength of 450 nm. This is likely due to the high photon energy of the near UV light (405 nm) damaging the chemical linkers and detaching the analyte from the sensor surface. Camera exposure time: 0.5 ms for the incident wavelength of 405 nm, and 2.5 ms for the incident wavelength of 450 nm.

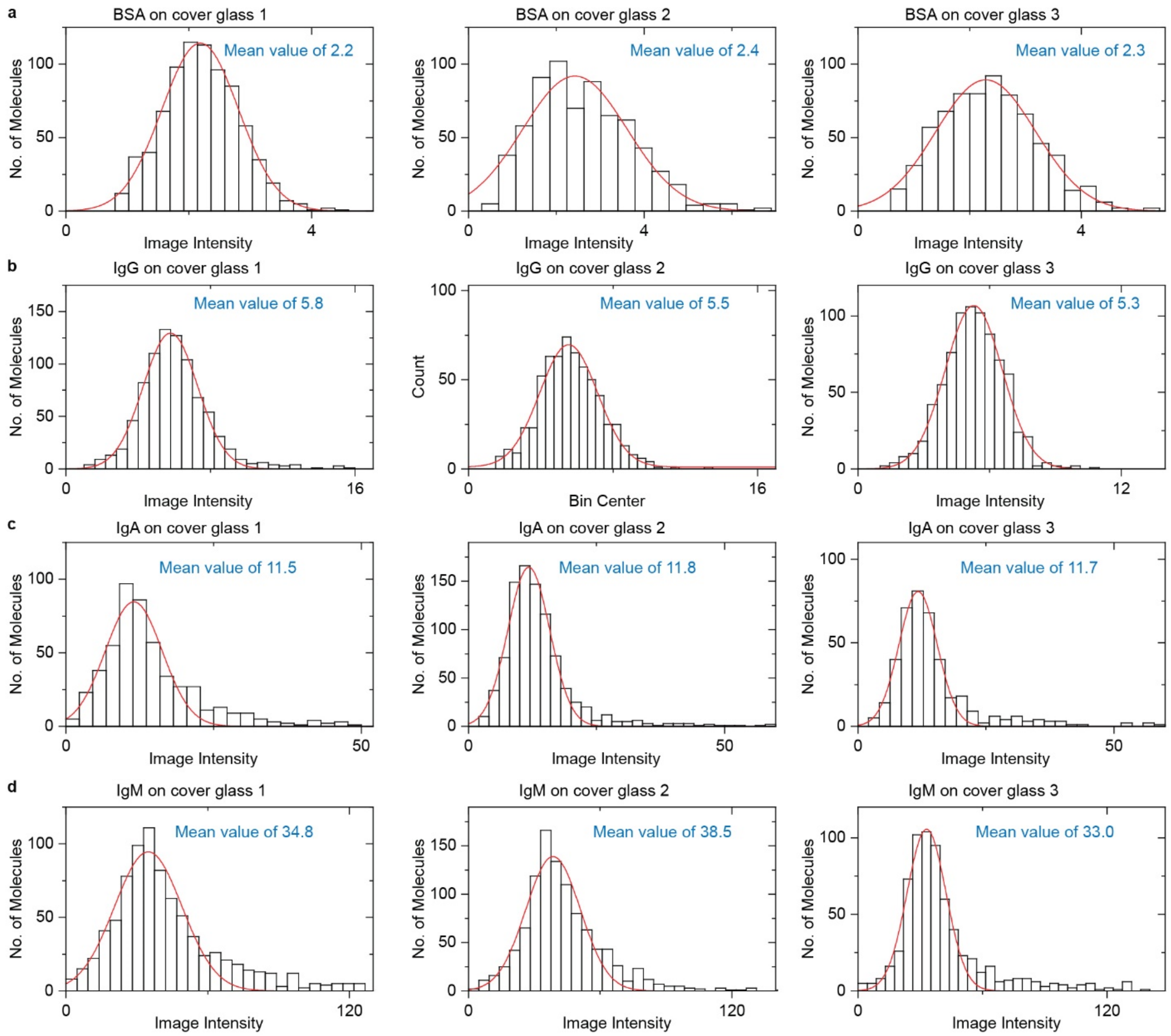

Supplementary Figure 10

ESM image intensity histograms of BSA (a), IgG (b), IgA (c), and IgM (d) proteins on different cover glasses.

The solid red curves are Gaussian fittings. The mean values of the corresponding histograms were employed as the image intensity for each protein shown in Figure 2e. The measurement results on cover glass No.1 were the same as those shown in Figure 2a-d. Incident wavelength: 450 nm. Incident intensity: 60 kW cm^{-2}. Camera exposure time: 5 ms.

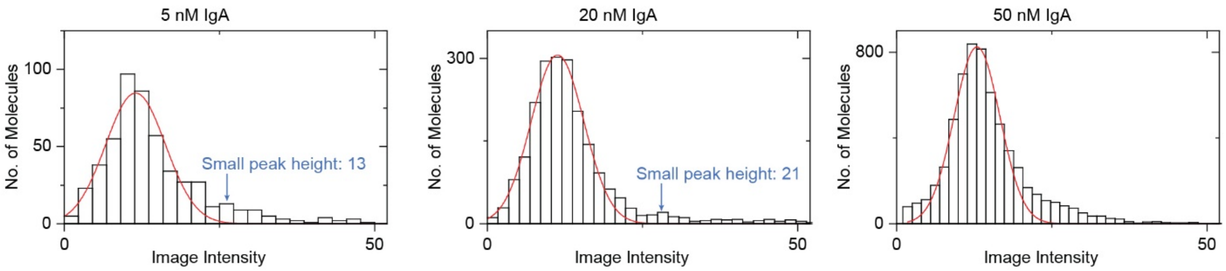

Supplementary Figure 11

ESM image intensity histograms measuring IgA protein with different solution concentrations.

The solid red curves are Gaussian fitting. The arrows indicate the minor peaks shown at the position with ~ two times higher intensity than the mean values. The peak height does not scale with the protein concentration, and becomes a tail at a high analyte concentration, indicating that they are not created by dimmers. The possible reason is that two or more molecules were simultaneously falling within the same Airy disk area with a diameter of ~ 1 µm, which cannot be resolved in the image and counted as a single binding event. Incident wavelength: 450 nm. Incident intensity: 60 kW cm^{-2}. Camera exposure time: 5 ms.

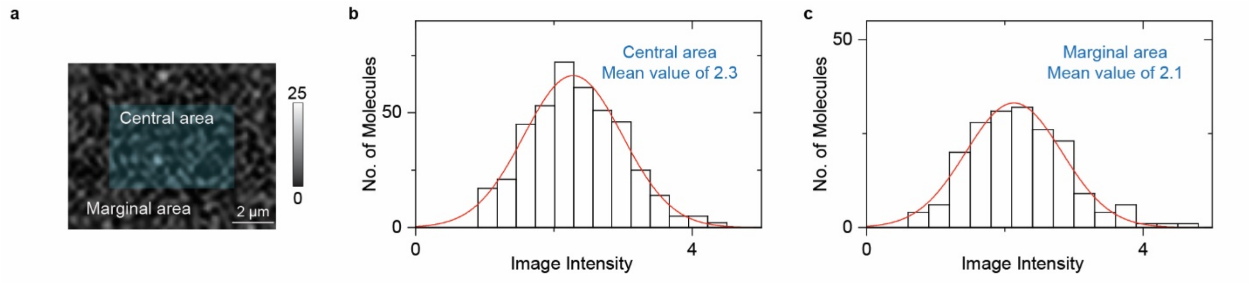

Supplementary Figure 12

ESM image intensity histograms measuring BSA proteins in different regions.

The BSA proteins recognized in the central area present slightly higher image intensity than those in the marginal area, and thus the proteins binding on the central illumination area will be recognized more easily. Incident wavelength: 450 nm. Incident intensity: 60 kW cm^{-2}. Camera exposure time: 5 ms.

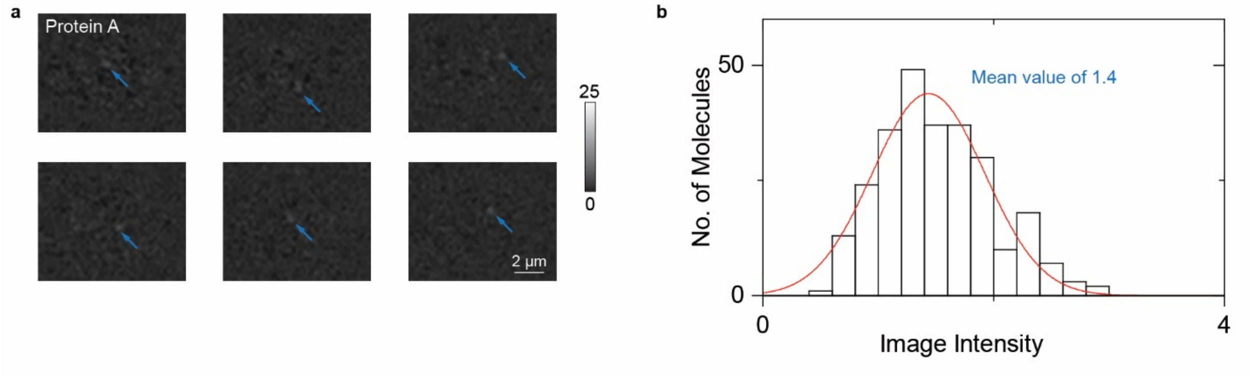

Supplementary Figure 13

ESM images and intensity histograms of protein A molecules.

The protein A proteins can be recognized in the central illumination area. The individual proteins are marked with arrows. Incident wavelength: 450 nm. Incident intensity: 60 kW cm^{-2}. Camera exposure time: 5 ms. The experiments were repeated three times with similar results.

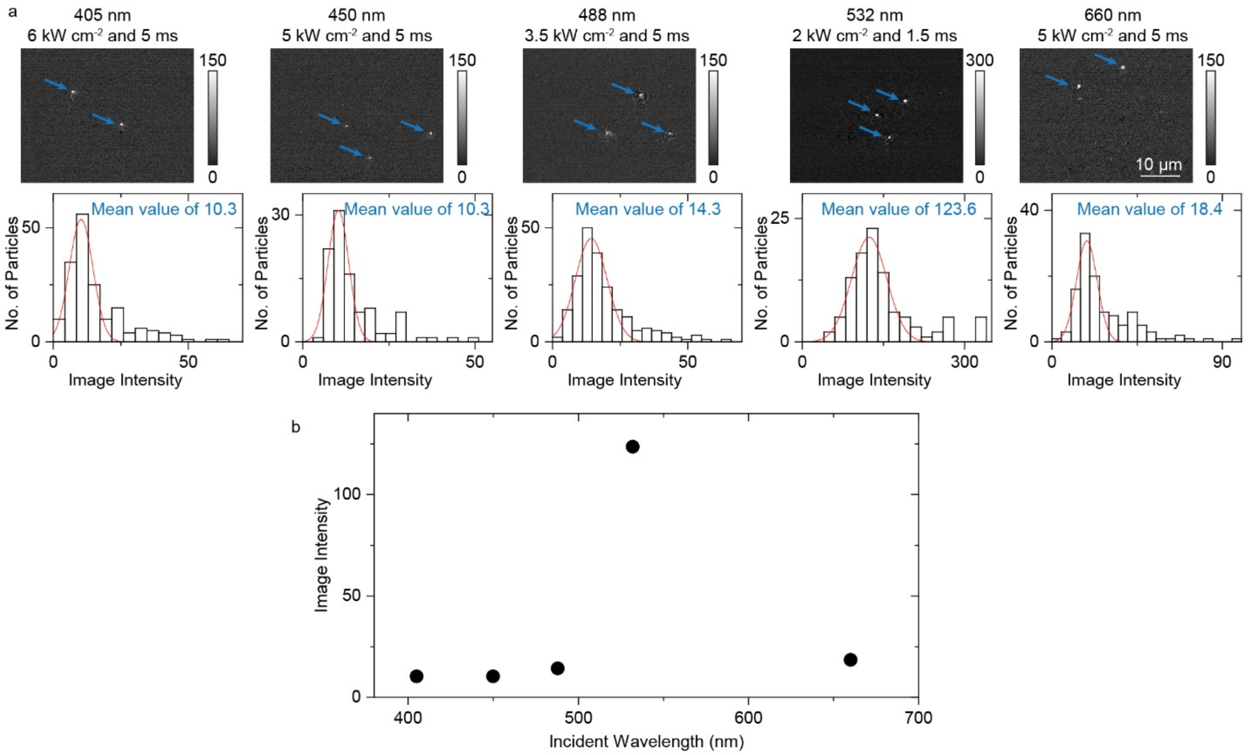

Supplementary Figure 14

(a) ESM images and intensity histograms of 30 nm gold nanoparticles under different incident wavelengths. The incident intensity and camera exposure time are presented. The image intensity was normalized with the incident intensity of 2 kW cm^{-2} and the camera exposure time of 1.5 ms. The experiments were repeated three times with similar results. (b) ESM image intensity of 30 nm gold nanoparticles versus incident wavelength, showing that the ESM incident intensity notably increases at the incident wavelength of 532 nm near the localized SPR peak of gold nanoparticles.

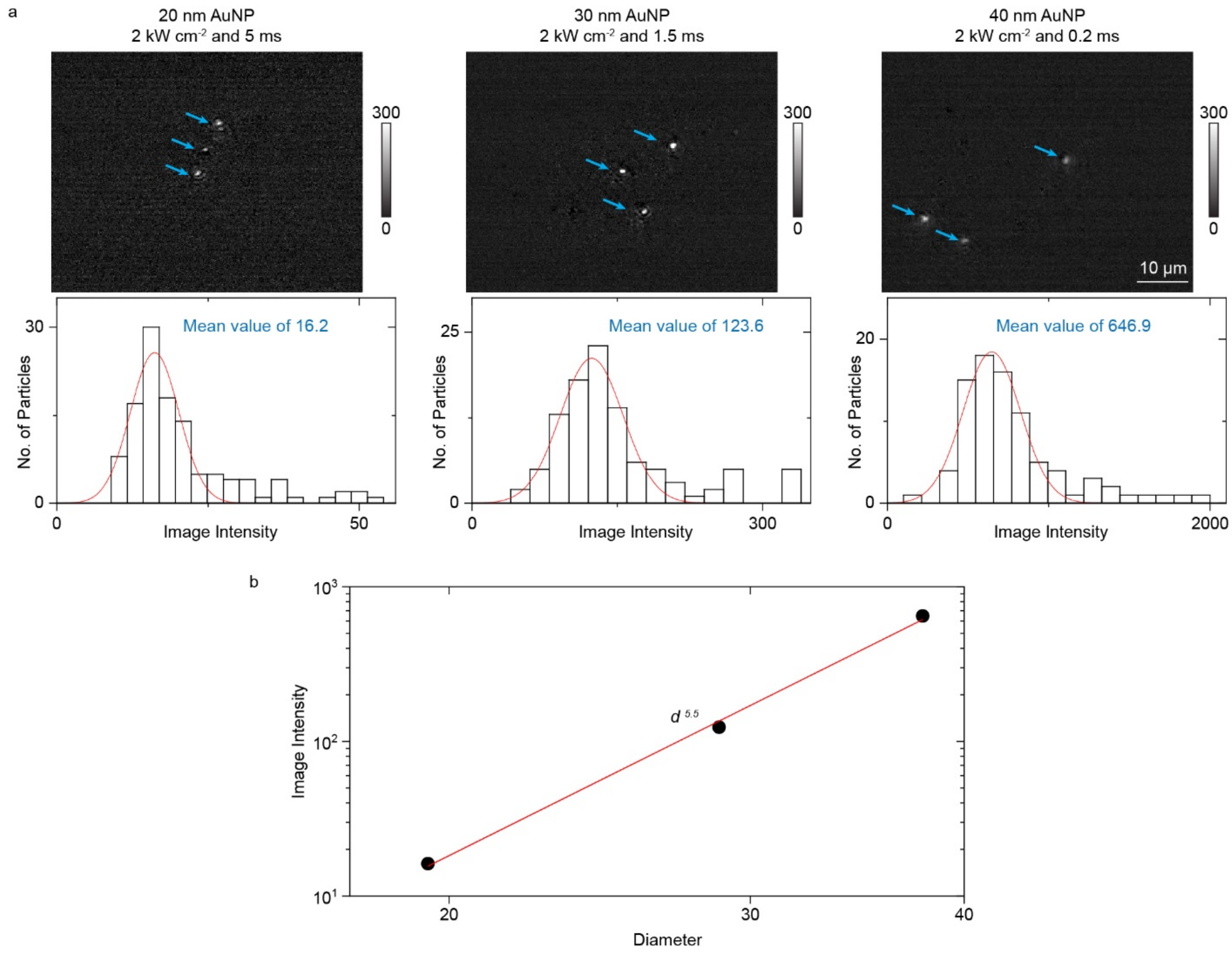

Supplementary Figure 15

(a) ESM images and intensity histograms of gold nanoparticles with different diameters at the incident wavelength of 532 nm. The image intensity was normalized with the incident intensity of 2 kW cm^{-2} and the camera exposure time of 1.5 ms. The experiments were repeated three times with similar results. (b) ESM image intensity versus gold nanoparticle diameter, where the z-distance dependence of evanescent wave is considered (Supplementary Note 1). The image intensity follows a power law of $d^{5.5}$, where the exponent is close to six, indicating that the nanoparticle scattering dominates the image intensity.

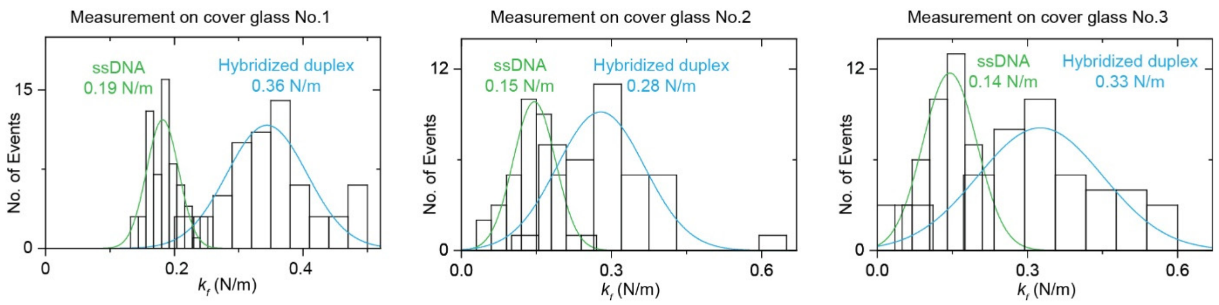

Supplementary Figure 16

Effective spring constant statistical distribution of gold nanoparticles linked by ssDNA molecules measured on three cover glasses before and after hybridization with miRNA, where the solid curves are Gaussian fitting. Glass No. 1 is shown in Figure 4i. Incident wavelength is 532 nm, and incident light intensity and camera exposure time are 2 kW cm^{-2} and 0.2 ms.

Supplementary Note 1

Effective diameter correction

The evanescent field decreases exponentially from the surface (z-direction) into the solution. In other words, the scattering of the evanescent field by a finite-size object depends on the distance (z) from the surface (Supplementary Figure 17). The effective scattering diameter D_{eff} and volume V_{eff} of the analyte can be given by

$$\frac{4}{3}\pi\left(\frac{D_{\text{eff}}}{2}\right)^3 = V_{\text{eff}} = \int_0^{2R} \pi\left(R^2 - (R - z)^2\right)e^{-\frac{z}{l}}\text{d}z = \int_0^{2R} \pi\left(Dz - z^2\right)e^{-\frac{z}{l}}\text{d}z, \quad \text{(N1.1)}$$

where z is the distance from the gold surface, R is the radius of the analyte, D is diameter of analyte, and l is the decay length of the evanescent field (PNAS, 2010, 107(37): 16028-16032).

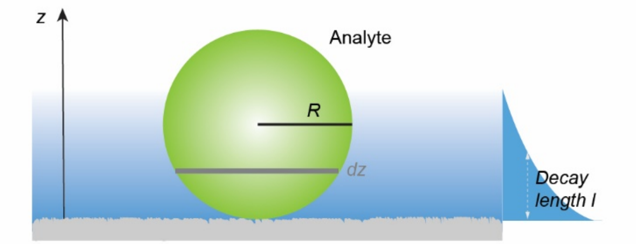

Supplementary Figure 17 Effective diameter correction model.

The decay length l of the evanescent field can be calculated by

$$l = \frac{\lambda}{4\pi\sqrt{n_{\text{glass}}^2\sin^2\theta - n_{\text{solution}}^2}}, \quad \text{(N1.2)}$$

where λ is the incident wavelength, θ is the incident angle, n_{glass} and $n_{solution}$ are the refractive indices of cover glass and solution, respectively (https://www.olympus-lifescience.com/en/microscope-resource/primer/java/tirf/penetration/). This study sets the incident angle at 65°. n_{glass} and $n_{solution}$ are 1.52 for BK7 cover glass and 1.33 for water, respectively. For an incident wavelength of 405 nm, 450 nm, 488 nm, 532 nm, and 660 nm, the decay length l of the evanescent field can be estimated to be 90 nm, 100 nm, 108 nm, 118 nm, and 146 nm, respectively. The polystyrene nanoparticles (PSNP) used in this study have diameters measured by dynamic light scattering of 27.9 nm, 41.3 nm, 61.6 nm, 93.7 nm, 143.6 nm. The bovine serum albumin (BSA), immunoglobulin G (IgG), immunoglobulin A (IgA), and immunoglobulin M (IgM) have diameters measured by dynamic light scattering of 8.5 nm, 11.8 nm, 15.7 nm, 21.8 nm, respectively. The effective diameter of the analyte under different incident wavelengths can be calculated by equation (N1.1) as shown in Supplementary Table N1.1. It is clear that the need for this correction decreases with the size, and the correction becomes insignificant for proteins.

Supplementary Table N1.1 Effective diameter of analyte in nanometer under different incident wavelengths.

Analyte diameter (nm)	Incident 405 nm	Incident 450 nm	Incident 488 nm	Incident 532 nm	Incident 660 nm
143.6 (PSNP)	112.4	115	116.8	118.7	122.9
93.7 (PSNP)	79.5	80.7	81.6	82.5	84.5
61.6 (PSNP)	55.2	55.8	56.2	56.6	57.5
41.3 (PSNP)	38.3	38.6	38.8	39	39.4
27.9 (PSNP)	26.5	26.6	26.7	26.8	27.0
21.8 (IgM)	21.0	21.0	21.1	21.1	21.3
15.7 (IgA)	15.3	15.3	15.3	15.4	15.4
11.8 (IgG)	11.6	11.6	11.6	11.6	11.7
8.5 (BSA)	8.4	8.4	8.4	8.4	8.4

Supplementary Note 2

Estimation with Rayleigh scattering model

The evanescent wave is one kind of subwavelength optical wave, owning more complex propagation and scattering properties than the far-field light (Nature Nanotech. 7, 668–672 (2012); Angew. Chem. Int. Ed. 58, 572 –576 (2019)). This complex behavior usually cannot be precisely described by current theory, while the numerical iteration and theoretical approximation analysis have shown that the object has larger scattering cross-section in the evanescent field than the far-field light field (Surface Science, 590, 173-180 (2005); Phys. Rev. B 92, 245419 (2015); Nat Commun 11, 4768 (2020)). Nevertheless, as a rough estimation, Rayleigh scattering model can be used to describe the evanescent scattering behaviors of small objects (Anal. Chem. 86, 8992–8997 (2014)).

Textbook shows that the Rayleigh scattering cross-section σ can be given by (https://en.wikipedia.org/wiki/Rayleigh_scattering)

$$\sigma = \frac{2\pi^5}{3}\frac{d^6}{\lambda^4}\left(\frac{\left(\frac{n_a}{n_m}\right)^2 - 1}{\left(\frac{n_a}{n_m}\right)^2 + 2}\right)^2, \qquad \text{(N2.1)}$$

where d is the analyte diameter, λ is the incident wavelength, and n_a and n_m are the refractive index of analyte and media, respectively. The media is water in this study. The equation (N2.1) shows that the scattering intensity of nanoparticle scales with λ^{-4}, close to the experimental results shown in Figure 1g.

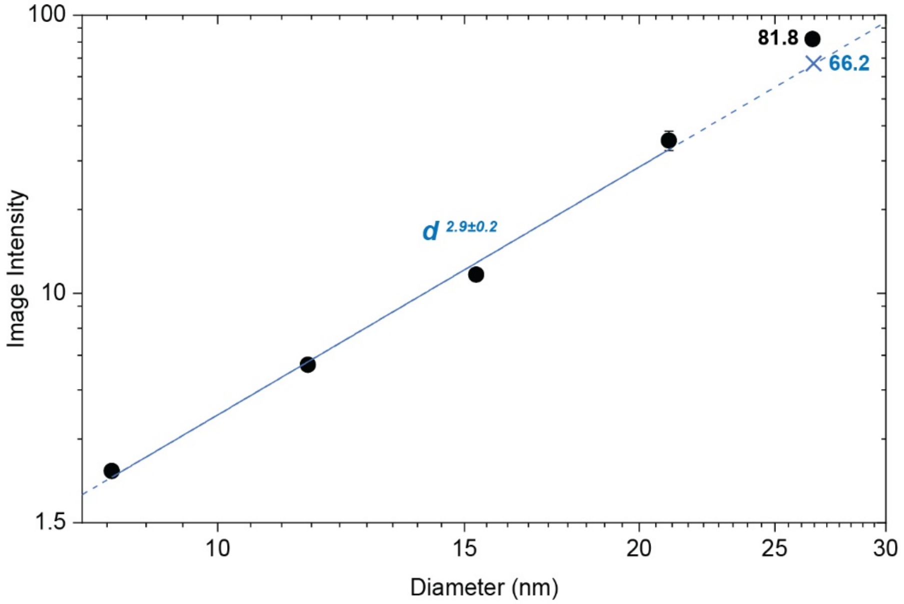

Supplementary Figure 18 ESM image intensity versus analyte diameters in logarithmic scale. The image intensities of BSA, IgG, IgA, and IgM were obtained from Figure 2a-d, and the image intensity of 27.9nm polystyrene nanoparticles were obtained from Supplementary Figures 5. The sample sizes have been marked in Figure 2a-d and Supplementary Figures 5. The error bars represent the standard deviations achieved from the image intensities determined by three independent measurements. The calibration curve was calculated from the image intensities of BSA, IgG, IgA, and IgM proteins. The proteins were measured on a carboxylic group modified surface, and polystyrene nanoparticles were measured on a poly-l-lysine modified surface. The cross sign indicates the image intensity of the protein with a diameter of 27.9 nm.

The experimental results show that the polystyrene nanoparticles present ~24% higher image intensity than proteins with the same diameter.

After configuring the incident wavelength and media, the Rayleigh scattering model shows that the analyte diameter and refractive index determine the scattering intensity. The polystyrene has a refractive index of 1.61 (refractiveindex.info) under the incident wavelength of 450 nm. On the other hand, the proteins in the evanescent field are reported to have a refractive index of 1.48 (Meas. Sci. Technol. 2006, 17, 932-938). Therefore, calculation with the Rayleigh scattering model shows that the protein scattering cross-section will equal ~30% of the scattering cross-section of polystyrene nanoparticles with the same diameter, leading to that the protein image intensity should be ~82% smaller than the image intensity of polystyrene nanoparticles with the same diameter under interference detection conditions. However, the experiments show that the ESM image intensities of proteins are only ~24% smaller than that of the polystyrene nanoparticles with the same diameter (Supplementary Figure 18). This discrepancy should be because of the different binding conditions. The polystyrene nanoparticles bind to the poly-l-lysine modified surface via electrostatic adsorption, and proteins bind to the carboxyl group modified surface via covalent bonding. The covalent bonding usually can ensure that the proteins stay tightly on the surface (J. Am. Chem. Soc. 2019, 141, 16071–16078), while the electrostatic absorption may just loosely absorb the objects, leading to smaller image intensity due to the exponentially decaying property of evanescent fields (Anal. Chem. 2010, 82, 234–240). Considering that antibody modified nanoparticles usually have complicated behaviors on the surface (J. Am. Chem. Soc. 2019, 141, 16071–16078), the ESM image intensities of proteins on the poly-l-lysine modified surface were measured for comparison with those of the 27.9 nm polystyrene nanoparticles (Supplementary Figure 19). The experimental results show that the polystyrene nanoparticles present ~80% higher image intensity than the proteins with the same diameter under the same binding conditions, agreeing with the theoretical predictions.

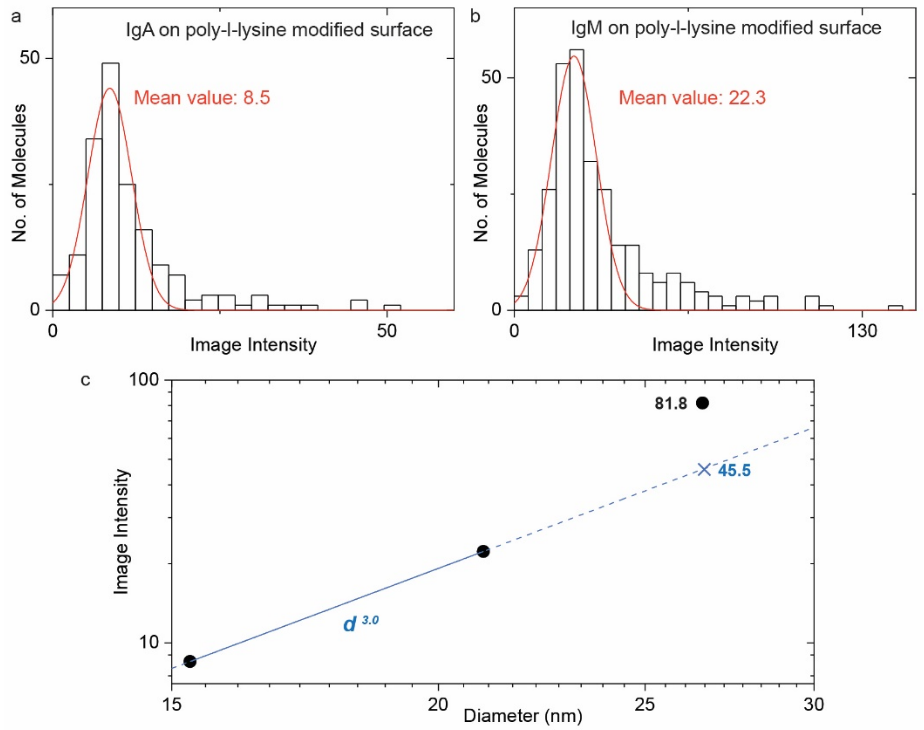

Supplementary Figure 19 ESM image intensity histograms of IgA (a), and IgM (b) on poly-l-lysine modified surface. (c), ESM image intensity versus analyte diameters in logarithmic scale. Image Intensities of IgA and IgM were obtained from (a) and (b), and image intensity of 27.9 nm polystyrene nanoparticles

is obtained from Supplementary Figure 5. The cross sign indicates the image intensity of the protein with a diameter of 27.9 nm estimated by image intensities of IgA and IgM. The experimental results show that the polystyrene nanoparticles present ~80% higher image intensity than the proteins with the same diameter.

It should be noted that the cubic power law of d^3, where d is the object diameter, achieved in Figure 2 does not conflict with the results shown for iSCAT or mass photometry. As shown in Supplementary Figure 20, we can also find that the ESM image intensity is proportional to the mass. We use diameter here to easily compare the ESM measurement results with the analyte diameter determined by the dynamic light scattering.

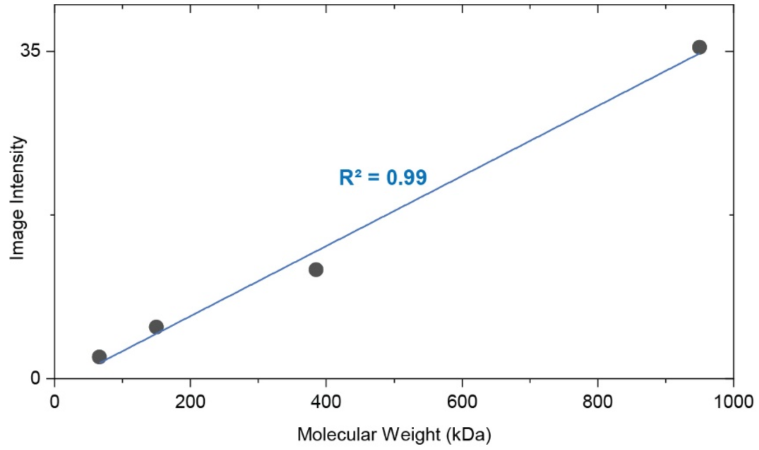

Supplementary Figure 20 ESM image intensity versus analyte molecular weight in linear scale. The image intensities of BSA, IgG, IgA, and IgM were obtained from Supplementary Figure 10.

The Rayleigh scattering model can also be used to estimate the system signal-to-noise ratio (SNR) theoretically. For the ESM measurement of single proteins, the image intensity I was determined by the interference between evanescent wave scattered by the surface roughness E_b and protein E_s as

$$I = |E_\mathrm{b}|^2 + 2|E_\mathrm{b}||E_\mathrm{s}|\cos(\theta) + |E_\mathrm{s}|^2, \tag{N2.2}$$

where θ is the phase difference between E_b and E_s.

In the shot noise dominant system, the SNR is determined by the shot noise, which can be shown as

$$SNR = \frac{2|E_\mathrm{b}||E_\mathrm{s}|\cos(\theta)}{\sqrt{|E_\mathrm{b}|^2 + 2|E_\mathrm{b}||E_\mathrm{s}|\cos(\theta) + |E_\mathrm{s}|^2}} \approx \frac{2|E_\mathrm{b}||E_\mathrm{s}|\cos(\theta)}{\sqrt{|E_\mathrm{b}|^2}} = 2|E_\mathrm{s}|\cos(\theta), \tag{N2.3}$$

The phase difference θ is about zero in ESM because of the short distance between scattering sites of surface roughness and analyte binding positions (ACS Photonics 8, 2227-2233 (2021)). The equation shows that the SNR is mainly determined by the scattering amplitude detected by the camera in the shot noise dominant system.

To estimate the theoretical SNR limit for the ESM system, the total Rayleigh scattering intensity I_{total} of one protein molecule is firstly estimated by

$$I_{\text{total}} = \frac{\frac{2\pi^5}{3} \times \frac{d^6}{\left(\frac{\lambda}{n_{\text{m}}}\right)^4} \times \left(\frac{\left(\frac{n_{\text{s}}}{n_{\text{m}}}\right)^2 - 1}{\left(\frac{n_{\text{s}}}{n_{\text{m}}}\right)^2 + 2}\right)^2}{A} \times P \times A \times t, \tag{N2.4}$$

where n_s and n_m are the refractive indices of analyte and medium, λ is the incident wavelength, d is the analyte diameter, P is the incident light intensity, and t is the average period. For the ESM used in this study, $n_s = 1.48$, $n_m = 1.33$, $\lambda = 450$ nm, and $t = 0.05$ s. Considering the single photon energy of ~1.2398/(0.45 µm) eV and the 5 x intensity enhancement of evanescent field, the total scattering intensity of one object in the ESM system can be expressed as

$$I_{\text{total}} = 5 \times 10^{-4} \times \left(d(\text{nm})\right)^6 \times P\ (\text{kWcm}^{-2})\ \text{photons}, \tag{N2.5}$$

The objective collects the scattering photons in perpendicular to the propagation direction of the evanescent wave, and the collection efficiency can be calculated with the equation in a spherical coordinate system of

$$\frac{I_{\text{collection}}}{I_{\text{total}}} = \frac{\int_{\theta_1}^{\theta_2}\int_{\varphi_1}^{\varphi_2} \frac{(1+\cos^2\theta)}{R^2} R^2 \sin\theta \mathrm{d}\varphi \mathrm{d}\theta}{\int_0^{\pi}\int_0^{2\pi} \frac{(1+\cos^2\theta)}{R^2} R^2 \sin\theta \mathrm{d}\varphi \mathrm{d}\theta}, \tag{N2.6}$$

where θ and φ are the polar angle and azimuthal angle, respectively. The objective collection angle for the ESM can be calculated by

$$\vartheta = \arcsin\left(\frac{NA}{n_{\text{m}}=1.33}\right), \tag{N2.7}$$

where NA is the objective numerical aperture, and $n_m = 1.33$ is used to correct the effect of water refraction on the scattering light collection. For the objective with an NA of 0.42, the collection efficiency is calculated to be ~2.5 %.

For the detection of BSA with a diameter of 8.4 nm under the incident intensity of 60 kW cm^{-2} and the average period of 50 ms, the total number of scattering photons is ~10000 based on equation (N2.5), and the number of photons collected by the objective is ~250 based on equations (N2.6) and (N2.7). Thus, the theoretical limit of SNR is ~ 30 under perfect conditions based on equation (N2.3). The transmission ratio of the imaging objective and tube lens in the ESM is measured to be ~70%, and the quantum efficiency of the XIEMA MQ003 camera is ~40% for the incident wavelength of 450 nm, so ~70 photoelectrons actually contribute to the sensor output. Considering ~40% higher noise brought by the differential processing, the shot noise limited SNR of our setup for the BSA detection with ESM employing the incident intensity of 60 kW cm^{-2} and the average period of 50 ms is ~ 10, agreeing with the experimental results.

Supplementary Note 3

Image Intensity calculation

The object usually creates bright spots with more complex patterns than conventional Airy patterns on the evanescent scattering images, which may be caused by the delocalization features of evanescent waves along the surface (Nat Methods 17, 1010–1017 (2020)). Supplementary Figure 21 shows that both ESM images of single 143.6 nm polystyrene nanoparticle (PSNP) and BSA protein have complex intensity distribution, especially at the marginal area. Therefore, we do not employ the two-dimensional Gaussian fitting to process the images but average the intensities of all pixels within the diffraction-limited area to produce the sensor output. The diameter of the diffraction-limited area was estimated to be ~1.07 µm by dividing the incident wavelength of 450 nm with the imaging objective numerical aperture of 0.42. The objective magnification is 50x, and the XIMEA MQ003 camera pixel size is 7.4 µm, so the diameter of a diffraction-limited area on the image can be estimated to be 1.07*50/7.4 ~ 7.2 pixels. To ensure taking all the pixels receiving the light into consideration, we employ the circle with a diameter of 8 pixels in the TrackMate v6.0.1 to measure the mean intensity of bright spots achieved by ESM.

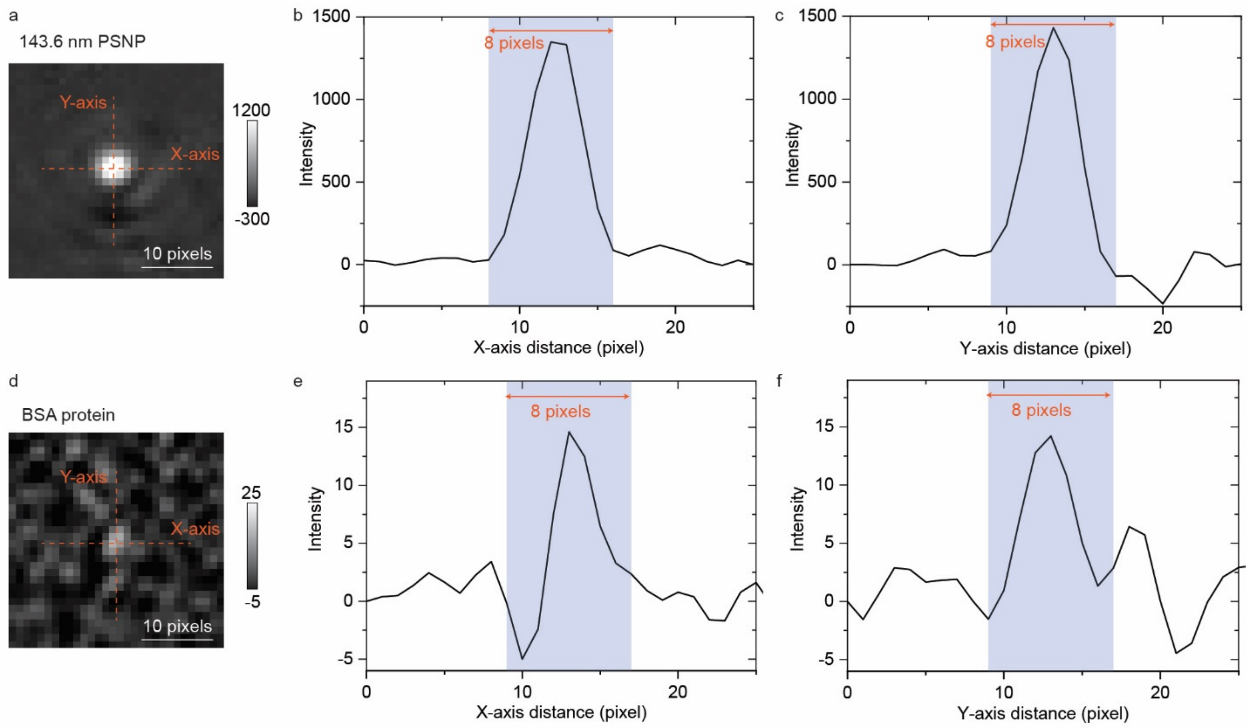

Supplementary Figure 21 (a) ESM image of one 143.6 nm polystyrene nanoparticle (PSNP). (b) & (c) Intensity profiles of marked lines at (a). Incident intensity: 2 kW cm^{-2}. Camera exposure time: 0.2 ms. (d) ESM image of one BSA protein. (e) & (f), Intensity profiles of marked lines at (a). Incident intensity: 60 kW cm^{-2}. Camera exposure time: 5 ms.

Supplementary Figure 21 shows that negative intensities exist within the diffraction-limited area. In addition, due to the finite size of camera pixels, the TrackMate v6.0.1 calculates the average value of intensities of 61 pixels with setting the circle diameter to be 8 pixels, where the total area should be closer to a square shape rather than a round shape, resulting in taking some blank pixels into the calculation. These two factors lead to the mean intensity being usually 5~7 smaller than the maximum intensity of the bright spots (Supplementary Figure 22). The ratio of maximum intensity to mean intensity fluctuates. This is likely

because the maximum intensity is a single-pixel value, and is subject to shot noise and camera pixel noise, especially for the weak signals with low signal to noise ratios, such as the measurement results of 27.9 nm and 41.3 nm polystyrene nanoparticles with an incident intensity of 2 kW cm^{-2} and BSA proteins with an incident intensity of 60 kW cm^{-2}.

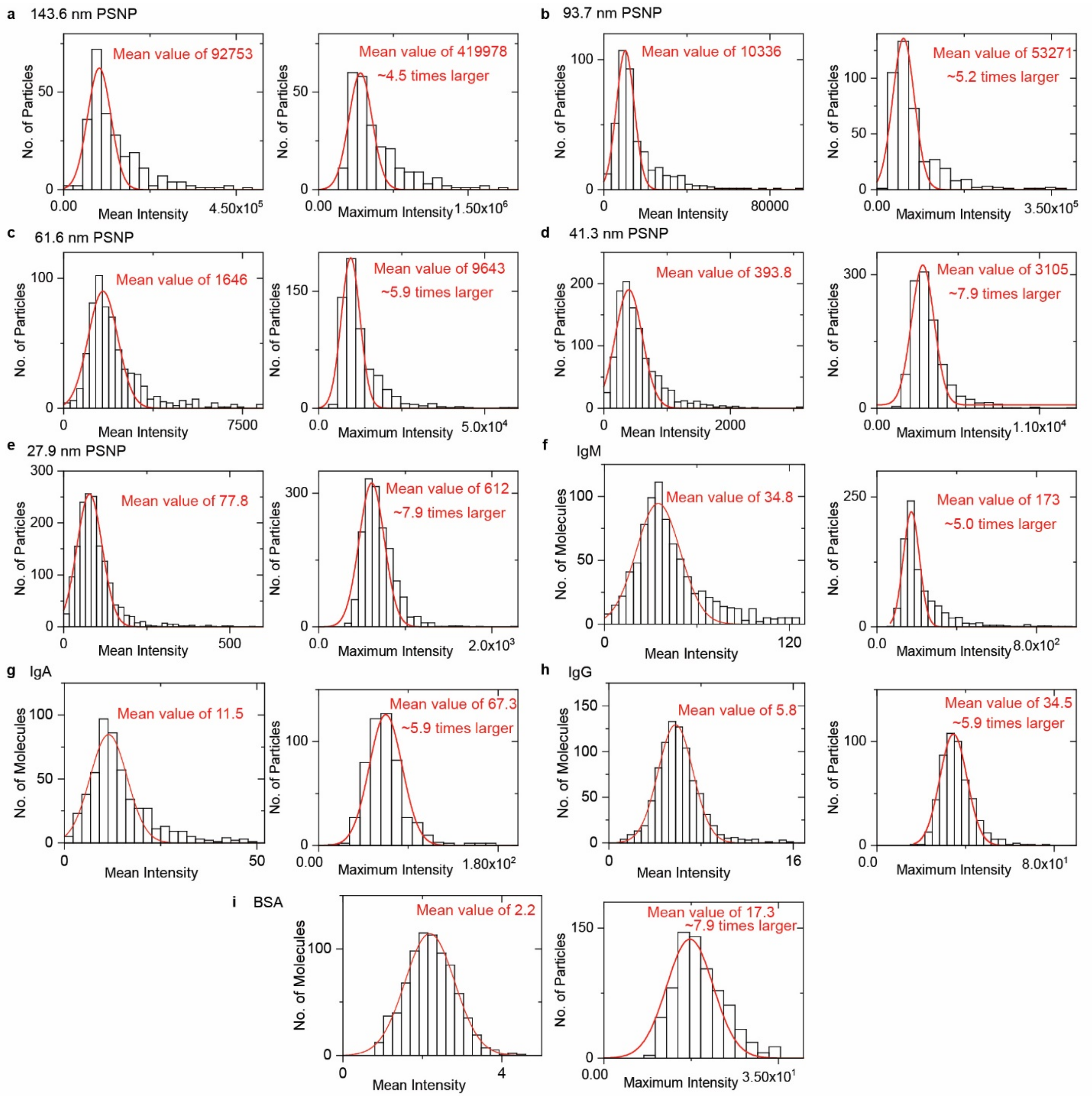

Supplementary Figure 22 Mean ESM image intensity, maximum ESM image intensity, and their ratio for 143.6 nm (a), 93.7 nm (b), 61.6 nm (c), 41.3 nm (d), and 27.9 nm (e) polystyrene nanoparticles (PSNP), and IgM (f), IgA (g), IgG (h), and BSA (i) proteins, where the solid red curves are Gaussian fittings. For PSNP: Incident intensity: 2 kW cm^{-2}. Camera exposure time: 5 ms for 27.9 nm, 2 ms for 41.3 nm and 61.6 nm, 1 ms for 93.7 nm, 0.2 ms for 143.6 nm. For proteins: Incident wavelength: 450 nm. Incident intensity: 60 kW cm^{-2}. Camera exposure time: 5 ms. The image intensity was normalized with an incident intensity of 60 kW cm^{-2} and a camera exposure time of 5 ms.

Supplementary Note 4

Tracking precision estimation

For single particle tracking, the tracking precision in one dimension can be theoretically described by

$$\sigma_{\mu i} = \sqrt{\frac{s_i^2}{N} + \frac{a^2}{12N} + \frac{8\pi s_i^4 b^2}{a^2 N^2}}, \tag{N4.1}$$

where $\sigma_{\mu i}$ is the standard deviation of localization positions at *i*-th dimension, N is the photo number, a is the pixel size/magnification, s is the standard deviation of Gaussian distribution, and b is the standard deviation of camera output in the dark (Science, 300, 2061-2065 (2003)). The photon number N was used to estimate the shot noise limited signal to noise ratio (SNR), which is equal to the square root of photon number. For interference detection, the photon number N can be represented by the square of the shot noise limited SNR (Nat Commun 5, 4495 (2014)). The full width at half maximum (FWHM) can be estimated for optical imaging by dividing the incident wavelength by two times of imaging objective numerical aperture (NA), and the standard deviation can be estimated by dividing the FWHM by 2.35 (https://en.wikipedia.org/wiki/Full_width_at_half_maximum). Thus, the s can be estimated by

$$s_i = \frac{\lambda}{2 \times 2.35 \times NA}, \tag{N4.2}$$

For the IgA measured under the incident intensity of 20 kW cm^{-2} and the average period of 20 ms, the SNR can be estimated to be ~15. The camera used in this study is XIMEA MQ003MG-CM with a pixel size of 7.4 μm, and the objective has NA of 0.42 and magnification of 50x. Thus, a can be estimated to be 0.148 μm. b can be measured to be ~0.8 photons. So, for incident wavelength of 0.45 μm, the equation N4.1 can be calculated as

$$\sigma_{\mu i} = \sqrt{\frac{0.45^2}{(2 \times 2.35 \times 0.42)^2 \times 15^2} + \frac{0.148^2}{12 \times 15^2} + \frac{8\pi \times 0.8^2}{0.148^2} \times \frac{0.45^4}{(2 \times 2.35 \times 0.42)^4 \times 15^4}}$$

$$= \sqrt{2.3 \times 10^{-4} + 8.1 \times 10^{-6} + 3.9 \times 10^{-5}} \approx 0.0166\ \mu\text{m} = 16.6\ \text{nm}, \tag{N4.3}$$

The equation N4.3 shows that the second and third terms are much smaller than the first term for ESM. Thus, the equation N4.1 can be simplified as

$$\sigma_{\mu i} \approx \frac{\lambda}{2 \times 2.35 \times NA \times SNR}, \tag{N4.4}$$

Supplementary Note 5

Relationship between the ESM image intensity and z-displacement

The relation between evanescent field intensity I_E and z-displacement can be given by (J. Am. Chem. Soc. 2019, 141, 40, 16071–16078)

$$I_{\mathrm{E}} = I_{\mathrm{E0}} e^{-z/l}, \tag{N5.1}$$

where I_{E0} is the evanescent field intensity at z = 0, and l is the decay length of the evanescent field, which is 100 nm, 118 nm for the incident wavelength of 450 nm, and 532 nm, respectively (Supplementary Note 1). Under pure scattering conditions, the z-displacement of the analyte can be estimated by

$$\Delta z = z(t+\Delta t) - z(t) = l \times \ln\left(\frac{I_{\mathrm{E0}}}{I_{\mathrm{E}}(t+\Delta t)}\right) - l \times \ln\left(\frac{I_{\mathrm{E0}}}{I_{\mathrm{E}}(t)}\right) = l \times \ln\left(\frac{I_{\mathrm{E}}(\mathrm{t})}{I_{\mathrm{E}}(t+\Delta t)}\right), \tag{N5.2}$$

Under interference conditions, the scattering amplitude E_S of the analyte can be estimated by

$$E_{\mathrm{s}} = \sqrt{I_{\mathrm{E}}} = \sqrt{I_{\mathrm{E0}}} e^{-z/2l}, \tag{N5.3}$$

So, the z-displacement of the analyte can be estimated by

$$\Delta z = z(t+\Delta t) - z(t) = 2l \times \ln\left(\frac{\sqrt{I_{\mathrm{E0}}}}{E_{\mathrm{s}}(\mathrm{t}+\Delta\mathrm{t})}\right) - 2l \times \ln\left(\frac{\sqrt{I_{\mathrm{E0}}}}{E_{\mathrm{s}}(\mathrm{t})}\right) = 2l \times \ln\left(\frac{E_{\mathrm{s}}(t)}{E_{\mathrm{s}}(t+\Delta t)}\right)$$
$$= 2l \times \ln\left(\frac{2E_{\mathrm{b}}E_{\mathrm{s}}(\mathrm{t})}{2E_{\mathrm{b}}E_{\mathrm{s}}(t+\Delta t)}\right), \tag{N5.4}$$

The statistical distribution of fluctuating z-displacement amplitude can be used for evaluating the free energy profiles of one binding pair according to

$$P(z) = A \exp\left[-\frac{G(z)}{k_{\mathrm{B}}T}\right], \tag{N5.5}$$

where A is a constant that can be determined by normalization of $P(z)$, k_B is the Boltzmann constant, and T is the temperature (PNAS 2003, 100 (20), 11378– 11381; PNAS 2020, 117 (44). 27148-27153). The effective spring constant k_f can be determined from the simplified expression of free energy profile near equilibrium shown as

$$G(z) = G(0) + \frac{1}{2}k_{\mathrm{f}}z^2, \tag{N5.6}$$

where $G(0)$ is the free energy at equilibrium (J. Am. Chem. Soc. 2019, 141, 40, 16071–16078).

For the interference measurement, the background intensity fluctuation in the region adjacent to the protein binding site was calculated (Supplementary Figure 23) and shows much smaller fluctuations, indicating that the IgM binding dominated this z-axis movement shown in Figure 4b.

For the gold nanoparticle tracking, the nanoparticle intensity is independent of background intensity, thus allowing higher tracking precision. To evaluate the tracking precision, the intensities of all nanoparticles recorded in each frame were averaged for averaging out the random thermal fluctuations. Although the average results should still contain some contributions from thermal fluctuations because the number of particles is finite, the fluctuation after average still reveals that the tracking precision is at least 0.04 nm. (Supplementary Figure 24).

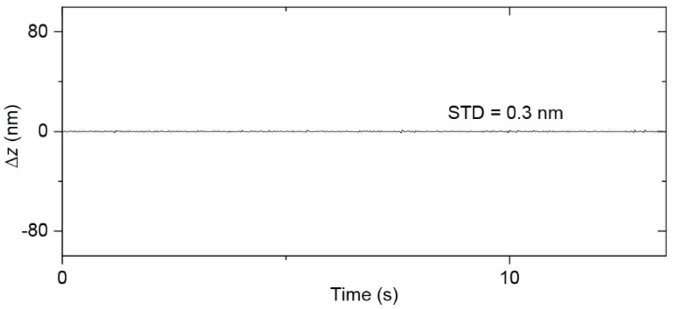

Supplementary Figure 23 Background intensity fluctuation in the region adjacent to the IgM binding event shown in Figure 4b.

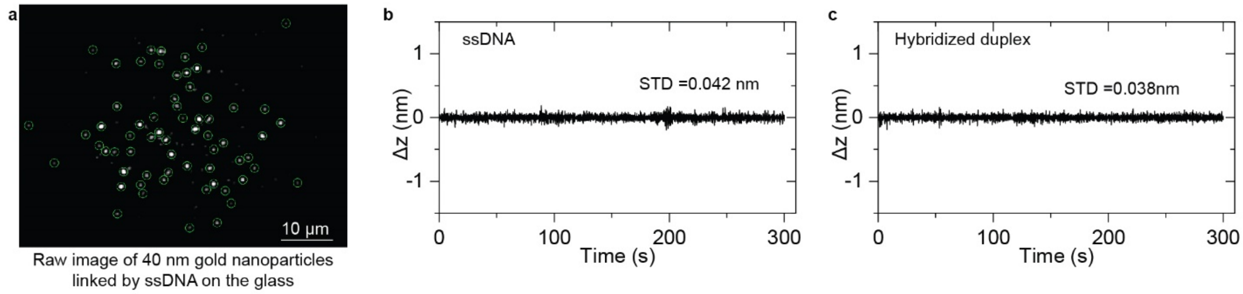

Supplementary Figure 24 (a) Raw images of 40 nm gold nanoparticles linked by ssDNA on the cover glass. The green circles mark the nanoparticles recognized and tracked for analyzing DNA conformation changes. (b), and (c) Thermal fluctuations achieved by averaging the image intensities of all nanoparticles on the cover glass before (b) and after (c) hybridization with complementary microRNA.

Supplementary Note 6

Force analysis on the DNA linker nanoparticles

Our flow cell's hydraulic diameter D_H can be estimated by

$$D_{\mathrm{H}} = \frac{2HW}{H+W} \approx \frac{2 \times 2 \times 0.05}{2 + 0.05} \approx 0.1 \text{ mm} \approx 10^{-4}\text{m}, \quad \text{(N6.1)}$$

where the width W is ~ 2 mm and height H is ~ 50 μm = 0.05 mm (https://en.wikipedia.org/wiki/Hydraulic_diameter). The Reynolds number Re can be estimated by

$$Re = \frac{\rho u D_{\mathrm{H}}}{\mu} = \frac{10^3 \times 3 \times 10^{-2} \times 10^{-4}}{10^{-3}} \approx 3, \quad \text{(N6.2)}$$

where ρ is the fluid density of ~10^3 kg/m^3, u is the mean flow rate of ~0.03 = 3× 10^{-2} m/s in the experiment with a volume flow rate of 200 μL/min, μ is the dynamic viscosity of ~10^{-3} Pa/s for PBS buffer (Nat. Chem. 13, 428–434 (2021)). Reynolds number is much smaller than 2300, indicating that the waterflow in our flow cell is laminar flow (https://en.wikipedia.org/wiki/Reynolds_number). The viscous damping force F_d can be estimated with Stoke's equation of

$$F_{\mathrm{d}} = 6\pi\eta r \frac{\mathrm{d}z}{\mathrm{d}t} = 6 \times \pi \times 10^{-3} \times 20 \times 10^{-9} \times \frac{0.3 \sim 1 \times 10^{-9}}{1 \times 10^{-1}} \approx 1.1 \sim 3.7 \times 10^{-6} \text{ pN}, \quad \text{(N6.3)}$$

where η is solvent viscosity (~10^3 Pa·s for water), r is the nanoparticle radius, the measurement duration dt is ~0.1 s for the vertical tracking of nanoparticle, and dz is the nanoparticle movement distance within the measurement duration, which is ~0.3 nm, and 1 nm for nanoparticles on hybridized duplex structure, and ssDNA, respectively (Figure 4e) (J. Am. Chem. Soc. 2019, 141, 40, 16071–16078).

The entropy force $F_{entropy}$ for the fully stretched ssDNA linker can be estimated with the FJC model, namely

$$F_{\mathrm{entropy}} = \frac{3k_{\mathrm{B}}T}{nb^2} z = \frac{3 \times 1.38 \times 10^{-23} \times (273.15 + 25)}{\left(\frac{b}{0.4}\right) \times \left(b \times 10^{-9}\right)^2} \times 0.4 \times 26 \times 10^{-9} = \frac{51}{b^3} \text{pN}, \quad \text{(N6.4)}$$

where b is the Kuhn length in nanometer, n is the number of segments with a length of b, 0.4 nm is the length of one base pair, 26 is the number of base pairs of the DNA used in this experiment (J. Chem. Phys. 142, 194902 (2015); Nat Commun 11, 4768 (2020)). ssDNA has the Kuhn length ranging from ~1.5 nm to ~8 nm (Science 271 (5250), 795-799 (1996); Macromolecules 30, 5763–5765 (1997)), and rigid dsDNA has Kuhn length of ~70 nm (J. Chem. Phys. 130, 215105 (2009)). The entropy force of fully stretched ssDNA linker before and after hybridization with microRNA can be estimated to be 0.1 to 15 pN and 10^{-4} pN, respectively.

The restoring force F_f of DNA linked nanoparticles can be estimated by

$$F_{\mathrm{f}} = k_{\mathrm{f}} z = 0.19 \times 1 \times 10^{-9} \sim 0.36 \times 0.3 \times 10^{-9} \approx 190 \sim 108 \text{ pN}, \quad \text{(N6.5)}$$

where k_f is the effective spring constant (Figure 4i), and z is nanoparticle movement distance (Figure 4e). These results clearly show that the restoring force resulting from the DNA linker is much larger than the viscous damping force and entropy force and should dominate the nanoparticle motions.

Supplementary Note7

Combination of ESM with fluorescence microscopy

A fluorescence illumination beam with a wavelength of 660 nm parallel to the surface was introduced to the ESM (Supplementary Figure 25a). The bottom objective collected the fluorescence signal with NA of 1.49 to image the labeled cells and released extracellular vesicles (Supplementary Figure 25b and c). One vesicle was captured by the BSA modified surface and showed rapid binding and unbinding behaviors (Supplementary Figure 25d), and its kinetic information can be analyzed with a single molecule binding kinetic analysis approach (Supplementary Figure 25e). ESM combined with fluorescence imaging may provide an approach for analyzing the surface property, which can help more accurately predict the diffusion mode of vesicles (Nat. Mater. 17, 740–746 (2018).

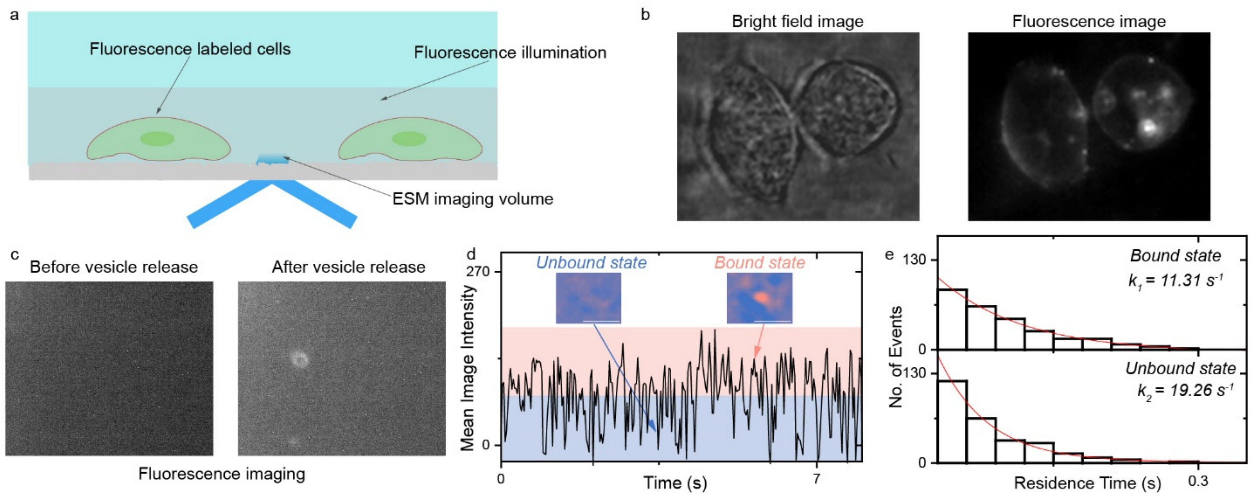

Supplementary Figure 25 (a) Schematic of the experimental setup. The evanescent field creates a small illumination volume for ESM. (b) Fluorescence labeled cells imaged by NA 1.49 objective. (c) Area adjacent to the cells imaged by fluorescence microscopy before and after extracellular vesicles release. (d) One vesicle was captured by the surface and showed rapid binding and unbinding behaviors. (e) Bound and unbound residence time distributions for the vesicle shown in (d), containing the information of kinetic between exosome and surface, which may indicate the surface property of exosome. Scale bar: 2 µm. The experiments were repeated three times with similar results.

Experimental details:

Materials. 0.01% sterile poly-l-lysine solution (Catalog No. P4707), potassium chloride (KCl, Catalog No. P9333), Magnesium chloride ($MgCl_2$, Catalog No. M2670), sodium chloride (NaCl, Catalog No. S7653), HEPES (Catalog No. H4034), glucose (Catalog No. G7021), calcium chloride ($CaCl_2$, Catalog No. C7902), serotonin (Catalog No. H9523), monoclonal anti-dinitrophenyl antibody (anti-DNP, Catalog No. D8406) were purchased from Sigma-Aldrich (St. Louis, MO, US). Heat inactivated fetal bovine serum (FBS, Catalog No. A3160602), DNP-BSA (Catalog No. A23018), Vybrant™ DiD cell-labeling solution (Catalog No. V22887), and 0.05% trypsin-EDTA (Catalog No. 25300054) were purchased from Thermo Scientific (Waltham, MA, US). RBL-2H3 cells (Catalog No. CRL-2256), and Eagle's Minimum Essential Medium (EMEM, Catalog No. 30-2003) were purchased from American Type Culture Collection (Manassas, VA, US). Penicillin-streptomycin (Catalog No. 30-002-CI) was purchased from Corning (Corning, NY, US).

Cell culture. RBL-2H3 cells were cultured in a humidity incubator at 37°C with 5% CO_2 and 70% relative humidity. EMEM with 10% FBS and 1% penicillin–streptomycin was used as a culture medium for cell growth. RBL-2H3 cells were cultured in a 25 cm^2 flask (Corning, Catalog No. 3289) and passaged when approximately 80% confluence was reached. 0.05% trypsin-EDTA was used for cell passage.

Vesicle Release Initiation. RBL-2H3 cells were transferred to a cover glass, which has been incubated in 0.01% sterile poly-l-lysine solution for 1 hour and rinsed with culture medium, for overnight culture. Before the experiment, 200 µM serotonin and 0.5 µg/mL anti-DNP were added to the culture medium for 1-hour incubation to prepare vesicle-release. Serotonin was used to increase the size of the vesicle, and the concentration that was used had no obvious effects on exocytosis kinetics. Then DiD solution was added into the culture medium for 15 min incubation to label the cell membranes. Anti-DNP was used to bind with the cell surface expressed high affinity IgE receptors. Then the cells were triple rinsed with 0.1% BSA in an extracellular buffer (135 mM NaCl, 5 mM KCl, 20 mM HEPES, 1.8 mM CaCl2, 1 mM MgCl2, 5.6 mM glucose, pH = 7.4) to block the surface followed by other triple washes with only extracellular buffer to remove extra BSA. A sample well with cells in 0.1 mL of extracellular buffer was transferred to the system, and ESM and fluorescent images were recorded after the addition of 10 µg/mL DNP-BSA, which was introduced to bind to anti-DNP and cross-link IgE receptors, resulting in a signaling cascade leading to vesicle exocytosis of the cells and release of chemical mediators.

Supplementary Note 8

MATLAB code for Differential processing

```
clear all;
warning off;
achieve=(‘file address\');
atarget=('file address \');
a=dir(achieve);
S=size(a);
pictotal=S(1)-2;
gap=1;
z=pictotal-gap;
for i=1:z
b1=a(i+2).name;
b2=a(i+2+gap).name;
path1=strcat(achieve,b1);
path2=strcat(achieve,b2);
I1=imread(path1);
I2=imread(path2);
A=double(I1);
B=double(I2);
C=B-A;
i_add=i;
i_str=sprintf('%07d',i_add);
path=strcat(atarget,strcat(i_str),'.tif');
imSave(path,single(C),'w');
end
```

Appendix (imSave function):

```
function [] = imSave(imName,img,mode)
t = Tiff(imName, mode);
tagstruct.ImageLength = size(img, 1);
tagstruct.ImageWidth = size(img, 2);
tagstruct.Compression = Tiff.Compression.None;
tagstruct.SampleFormat = Tiff.SampleFormat.IEEEFP;
tagstruct.Photometric = Tiff.Photometric.MinIsBlack;
tagstruct.BitsPerSample = 32;
tagstruct.SamplesPerPixel = 1;
tagstruct.PlanarConfiguration = Tiff.PlanarConfiguration.Chunky;
t.setTag(tagstruct);
t.write(img);
t.close();
end
```
