# Supplementary material for: Evanescent scattering imaging of single protein binding kinetics and DNA conformation changes
Source: Nat Commun. 2022 Apr 28;13:2298. doi: 10.1038/s41467-022-30046-8 (PMC9051210; doi:10.1038/s41467-022-30046-8)
Supplement: Supplementary file 3 — Description of Additional Supplementary Files [file 41467_2022_30046_MOESM3_ESM.pdf]

Title: Supplementary Video 1:
Description: Dynamic binding process of single proteins over time in the same grayscale.

Title: Supplementary Video 2:
Description: Exposure of anti-IgA modified sensor surface to IgA, Tg, and IgM proteins.

Title: Supplementary Video 3:
Description: Behaviours of one IgM molecule absorbed on the anti-IgM modified sensor surface.
