# Supplementary material for: Evanescent scattering imaging of single protein binding kinetics and DNA conformation changes
Source: Nat Commun. 2022 Apr 28;13:2298. doi: 10.1038/s41467-022-30046-8 (PMC9051210; doi:10.1038/s41467-022-30046-8)
Supplement: Supplementary file 7 — Reporting Summary [file 41467_2022_30046_MOESM7_ESM.pdf]

nature portfolio

Corresponding author(s): Shaopeng Wang

Last updated by author(s): Feb 25, 2022

Reporting Summary

Nature Portfolio wishes to improve the reproducibility of the work that we publish. This form provides structure for consistency and transparency in reporting. For further information on Nature Portfolio policies, see our Editorial Policies and the Editorial Policy Checklist.

Statistics

For all statistical analyses, confirm that the following items are present in the figure legend, table legend, main text, or Methods section.

n/a	Confirmed	
☐	☒	The exact sample size (n) for each experimental group/condition, given as a discrete number and unit of measurement
☐	☒	A statement on whether measurements were taken from distinct samples or whether the same sample was measured repeatedly
☒	☐	The statistical test(s) used AND whether they are one- or two-sided<br>*Only common tests should be described solely by name; describe more complex techniques in the Methods section.*
☒	☐	A description of all covariates tested
☒	☐	A description of any assumptions or corrections, such as tests of normality and adjustment for multiple comparisons
☐	☒	A full description of the statistical parameters including central tendency (e.g. means) or other basic estimates (e.g. regression coefficient) AND variation (e.g. standard deviation) or associated estimates of uncertainty (e.g. confidence intervals)
☒	☐	For null hypothesis testing, the test statistic (e.g. F, t, r) with confidence intervals, effect sizes, degrees of freedom and P value noted<br>*Give P values as exact values whenever suitable.*
☒	☐	For Bayesian analysis, information on the choice of priors and Markov chain Monte Carlo settings
☒	☐	For hierarchical and complex designs, identification of the appropriate level for tests and full reporting of outcomes
☒	☐	Estimates of effect sizes (e.g. Cohen's d, Pearson's r), indicating how they were calculated

Our web collection on statistics for biologists contains articles on many of the points above.

Software and code

Policy information about availability of computer code

Data collection	Data collection was performed using commercial software XIMEA CamTool Version 4.16.
Data analysis	Data analysis was performed using custom codes written in MATLAB R2018. The source codes are provided in Supplementary Note 8 and https://github.com/ZXY1231/PSMCellCode. TrackMate Plugin V6.0.1 in ImageJ (Fiji, v1.53c) was used for motion track. Origin 2019 was used to create data plots and histograms. Scrubber 2.0a was used to determine the association and dissociation rate constants by fitting curves with the first binding kinetics model.

For manuscripts utilizing custom algorithms or software that are central to the research but not yet described in published literature, software must be made available to editors and reviewers. We strongly encourage code deposition in a community repository (e.g. GitHub). See the Nature Portfolio guidelines for submitting code & software for further information.

Data

Policy information about availability of data

All manuscripts must include a data availability statement. This statement should provide the following information, where applicable:

- Accession codes, unique identifiers, or web links for publicly available datasets
- A description of any restrictions on data availability
- For clinical datasets or third party data, please ensure that the statement adheres to our policy

Source data of figures 1-4 are provided. The raw data that support the findings of this study are available from the corresponding author upon request.

March 2021

Field-specific reporting

Please select the one below that is the best fit for your research. If you are not sure, read the appropriate sections before making your selection.

☒ Life sciences ☐ Behavioural & social sciences ☐ Ecological, evolutionary & environmental sciences

For a reference copy of the document with all sections, see nature.com/documents/nr-reporting-summary-flat.pdf

Life sciences study design

All studies must disclose on these points even when the disclosure is negative.

Sample size	No sample size calculation was performed. We collected hundreds of nanoparticles or proteins to generate each histogram, and the experiments were reproduced by at least three times on different sensor chips. Hundreds of particles/molecules are sufficient to generate histograms to analyze the mean image intensity and compare it with theoretical predictions.
Data exclusions	No data point was excluded.
Replication	Detection of single nanoparticles and proteins were reproduced by at least 3 times on different sensor chips. All results are consistent and reproducible.
Randomization	All individual proteins and nanoparticles in the samples were randomly hitting the sensor surface and measured.
Blinding	Investigators were not blinded to group allocation as the statistical comparison between experimental groups was not part of the experimental design.

Reporting for specific materials, systems and methods

We require information from authors about some types of materials, experimental systems and methods used in many studies. Here, indicate whether each material, system or method listed is relevant to your study. If you are not sure if a list item applies to your research, read the appropriate section before selecting a response.

Materials & experimental systems

n/a	Involved in the study	
☐	☒	Antibodies
☐	☒	Eukaryotic cell lines
☒	☐	Palaeontology and archaeology
☒	☐	Animals and other organisms
☒	☐	Human research participants
☒	☐	Clinical data
☒	☐	Dual use research of concern

Methods

n/a	Involved in the study	
☒	☐	ChIP-seq
☒	☐	Flow cytometry
☒	☐	MRI-based neuroimaging

Antibodies

Antibodies used	Anti-human IgA is purchased from BioRad, catalog #STAR 141. Monoclonal anti-dinitrophenyl antibody was purchased from Sigma-Aldrich, Catalog No. D8406.
Validation	The binding specificity of Anti-human IgA antibody was validated by a control experiment (Fig. 3). The binding specificity of monoclonal anti-dinitrophenyl antibody was validated by fluorescence imaging, and the results have been published in ACS Sens. 2021, 6, 11, 4244–4254.

Eukaryotic cell lines

Policy information about cell lines

Cell line source(s)	RBL-2H3 was purchased from ATCC, catalog No. CRL-2256.
Authentication	None of the cell line used was authenticated.
Mycoplasma contamination	The cell line has been tested for mycoplasma contamination and the results are all negative.
Commonly misidentified lines (See ICLAC register)	No commonly misidentified cell lines in this study.
